# Systematic simulation of the interactions of pleckstrin homology domains with membranes

**DOI:** 10.1126/sciadv.abn6992

**Published:** 2022-07-06

**Authors:** Kyle I. P. Le Huray, He Wang, Frank Sobott, Antreas C. Kalli

**Affiliations:** 1 School of Molecular and Cellular Biology, Faculty of Biological Sciences, University of Leeds, Leeds, UK.; 2 Astbury Centre for Structural and Molecular Biology, Faculty of Biological Sciences, University of Leeds, Leeds, UK.; 3 Leeds Institute of Cardiovascular and Metabolic Medicine, School of Medicine, University of Leeds, Leeds, UK.; 4 School of Computing, University of Leeds, Leeds, UK.

## Abstract

Pleckstrin homology (PH) domains can recruit proteins to membranes by recognition of phosphatidylinositol phosphate (PIP) lipids. Several family members are linked to diseases including cancer. We report the systematic simulation of the interactions of 100 mammalian PH domains with PIP-containing membranes. The observed PIP interaction hotspots recapitulate crystallographic binding sites and reveal a number of insights: (i) The β1 and β2 strands and their connecting loop constitute the primary PIP interaction site but are typically supplemented by interactions at the β3-β4 and β5-β6 loops; (ii) we reveal exceptional cases such as the Exoc8 PH domain; (iii) PH domains adopt different membrane-bound orientations and induce clustering of anionic lipids; and (iv) beyond family-level insights, our dataset sheds new light on individual PH domains, e.g., by providing molecular detail of secondary PIP binding sites. This work provides a global view of PH domain/membrane association involving multivalent association with anionic lipids.

## Teaser

Simulating the binding of 100 pleckstrin homology domains to cell membranes reveals patterns in their lipid interactions.

## INTRODUCTION

Peripheral membrane proteins (PMPs) are proteins that transiently associate with the surface of cellular or organelle membranes (*1*, *2*). Binding of PMPs to membranes is often stabilized through a combination of specific and nonspecific interactions with the lipid headgroups, insertion of hydrophobic regions of the protein into the membrane interior and/or the presence of post-translational modifications that can anchor the protein to the membrane. There are several families of structurally conserved protein domains whose members have been identified as membrane-binding domains in PMPs. These families include C2 domains, Phox homology (PX) domains, FYVE domains, PDZ domains, and pleckstrin homology (PH) domains (*3*). Despite increasing structural and functional data about membrane-binding domains, knowledge of their membrane-binding interfaces, mechanism of association to the membrane, and whether there is any common mechanism of association at the family level remain elusive. Some members of these families are capable of recognizing specific lipid species, such as phosphatidylinositol phosphates (PIPs). PIPs are a minority lipid component in membranes, but they play a substantial role in the regulation of membrane protein activity and cellular signaling (*4*–*6*).

**Fig. 1.**
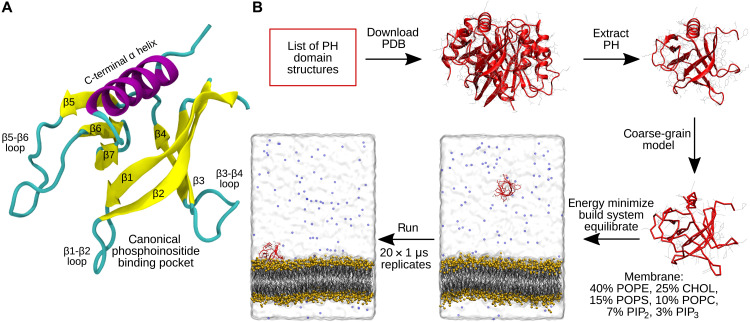
Conserved PH domain structure and simulation workflow. (**A**) Structure of the first PH domain of PLEK (PDB: 1xx0) demonstrates the conserved PH domain fold, consisting of a seven stranded β-barrel, capped by an α-helix, and with six variable interstrand loops. The open end of the barrel contains the canonical pocket for phosphoinositide binding. (**B**) Illustration of the semi-automated simulation pipeline used for high throughput PH domain simulations in this study.

PH domains are a large domain family with structural data available for more than 100 mammalian members. PH domains have a conserved fold (Fig. 1), which consists of a seven-stranded β-barrel, capped at one end by a C-terminal α-helix, with a pocket at the open end that is typically positively charged for interaction with anionic PIP headgroups. The PH domain of phospholipase Cδ1 (PLCδ1) was the first identified domain capable of specific binding to PIP lipids [phosphatidylinositol 4,5-bisphosphate (PIP_2_) in particular], and membrane localization mediated via specific binding to PIPs is the most studied characteristic of PH domains (*7*). Some family members are known to participate in regulatory protein-protein interactions with other proteins, leading to the proposal that some PH domains do not have a membrane-binding role (*8*, *9*). However, the most recent literature indicates that most of the family members localize to membranes and interact specifically with phosphoinositides (*10*, *11*).

Because of their crucial role in the regulation and activity of many signaling proteins, PH domains are implicated in a number of diseases. One example is the kinase Akt1 (RAC-alpha serine/threonine-protein kinase), whose activity is contingent on membrane localization mediated by its PH domain. Akt1 belongs to the most commonly activated proliferation signaling pathway in cancer, and the E17K mutation located in the canonical PIP binding pocket of the PH domain is oncogenic, as it increases membrane affinity and therefore Akt1 activity (*12*). Furthermore, in aberrant signaling proteins where the PH domain itself is not the locus of disease, targeting PH domain–membrane interactions has been shown to be a viable route to modulating the activity of the protein (*13*). Other PH domains with known links to disease include the PDPK1 (3-phosphoinositide–dependent protein kinase 1), P-Rex1 [phosphatidylinositol 3,4,5-trisphosphate-dependent Rac exchanger 1 protein], and IQSEC1 (IQ motif and SEC7 domain–containing protein 1) PH domains in cancer and intellectual disability; the BTK (Bruton’s tyrosine kinase) PH domain in autoimmune disease and X-linked agammaglobulinemia; and the FGD1 (FYVE, RhoGEF, and PH domain–containing protein 1) PH domain in faciogenital dysplasia (*12*, *14*–*20*). Consequently, there is interest in the development of small-molecule inhibitors of the PH domain–membrane interactions of these proteins, including recent work on inhibitors of P-Rex1 and IQSEC1 (*13*, *21*–*23*). Considering their importance in human disease and the pharmacological interest in mammalian PH domains, it is important to improve our understanding of their interactions with membranes. Many structures of PH domains that have been solved in complex with PIP lipid headgroups or suitable analogs typically demonstrate phosphoinositide binding at a so-called canonical site inside the pocket at the open end of the barrel (Fig. 1) (*15*, *24*–*26*). In this pocket, the headgroup phosphates are stabilized by electrostatic and hydrogen-bonded interactions with the strands and unstructured loops flanking the cavity. The importance of basic residues in the loop region connecting the β1 and β2 strands (the β1-β2 loop; Fig. 1) for the interaction with PIPs has been shown, and a KX_n_(K/R)XR sequence motif in this region has been identified as a predictor of binding to PIPs phosphorylated at the 3 position (*27*). However, phosphoinositide binding at atypical sites on the exterior of the barrel (fig. S1) and by PH domains lacking this sequence motif has also been observed, for example, in the case of the ArhGAP9 and β-spectrin PH domains (*28*, *29*).

A structure of the ASAP1 PH domain revealed dual binding of anionic lipids to both canonical and alternate sites simultaneously (*30*). A combination of simulations and experiments later revealed that multiple anionic lipid binding maintains the ASAP1 PH domain in an orientation conducive for interaction with its membrane-bound protein target (*31*). Furthermore, recent evidence has been presented for three PIP interacting sites on the PLEKHA7 PH domain and for PIP clustering induced by this PH domain (*32*). Similarly, an additional atypical site for soluble inositol hexakisphosphate binding has been observed in the BTK PH domain, which is critical for BTK activation (*33*). Subsequent simulations showed that multiple PIP binding sites stabilized dimerization of the BTK PH domain on the membrane (*34*). Additional sites for interaction with PIPs or anionic phosphatidylserine (PS) lipids have been identified in the Akt1, GRP1, PDK1, and BRAG2 PH domains (*35*–*40*). Furthermore, a large study of binding of yeast PH domains to liposomes of different composition demonstrated cooperative lipid binding in 93% of the liposome-binding PH domains (*10*). This growing body of evidence points to a new paradigm for PH domain membrane association, involving multivalent association with PIPs and other anionic lipids, rather than the one-to-one interaction mode suggested previously. It may be the case that the additional binding sites are weaker, more disordered, or too dependent on the membrane environment to be resolved by crystallography. Despite these recent data, however, it remains unclear how widespread the capacity for multiple PIP binding is throughout the family of mammalian PH domains.

Molecular dynamics (MD) simulations of membrane protein structures computationally reembedded into a lipid bilayer provide an excellent complement to experimental techniques and have proven to be a powerful tool for the identification of specific protein-lipid interaction sites (*41*, *42*). In particular, previous simulations of the membrane interactions of 13 PH domains whose structures had been solved in complex with PIP headgroups or analogs found that such simulations can identify the crystallographic PIP binding sites while also highlighting putative alternative sites of PIP interaction not revealed in the structures (*43*).

In this work, we simulated 100 mammalian PH domains, with the goal of establishing patterns in PH domain interactions with membranes at the family level. This large dataset additionally provides molecular detail for the 100 PH domains individually, which can be used to understand the mechanism of their association with the membrane, including new insights that we detail for some examples. We find that the PH domain β1 and β2 strands and their connecting loop contain the primary contact site for PIP headgroups in 85% of the analyzed PH domains, and most of those have frequent contacts with PIPs at alternative sites, such as β3-β4 and β6-β7 regions. Our analysis highlights the diversity of PH domain membrane interactions, and we have identified interesting exceptional cases. Furthermore, close association of multiple PIPs with the PH domains and clustering of PIPs induced by PH domain binding were universally observed in our simulations, with some PH domains exhibiting this to a greater extent than others.

## RESULTS

### A semi-automated pipeline for CG-MD simulation of PH domains

To perform simulations on this scale, we developed a semi-automated simulation pipeline (Fig. 1B). Given a PH domain–containing structure, it will extract the PH domain, remodel any missing atoms or residues, convert it to a coarse-grained (CG) representation, and then energy-minimize the CG model. The CG PH domain is initially placed in a water box at a 6-nm *z*-axis distance from a symmetric lipid bilayer model composed of 10% 1-palmitoyl-2-oleoyl-glycero-3-phosphocholine (POPC), 40% 1-palmitoyl-2-oleoyl-sn-glycero-3-phosphoethanolamine (POPE), 15% 1-palmitoyl-2-oleoyl-sn-glycero-3-phosphoserine (POPS), 7% phosphatidylinositol bisphosphate (PIP_2_), 3% phosphatidylinositol trisphosphate (PIP_3_), and 25% cholesterol. Further energy minimization and equilibration are conducted, after which, for each PH domain, 20 × 1 μs replicate production simulations are conducted, each initialized with different velocities. The protein explores many different orientations before binding to the bilayer, and thus, the initial orientation does not bias the subsequent membrane binding. We find that 20 replicates were sufficient for convergence in contact analysis and protein-membrane distance analysis (fig. S2). We note that the protein was placed outside the cutoff distance of the electrostatics of the membrane, which ensures that the protein does not experience forces from the bilayer at the beginning of the simulation. Not all simulation replicates resulted in stable membrane association, but membrane association was observed in most of the replicates for all simulated PH domains (fig. S3).

### Simulated phosphoinositide interaction sites are consistent with available crystal structures and suggest additional interaction sites

**Fig. 2.**
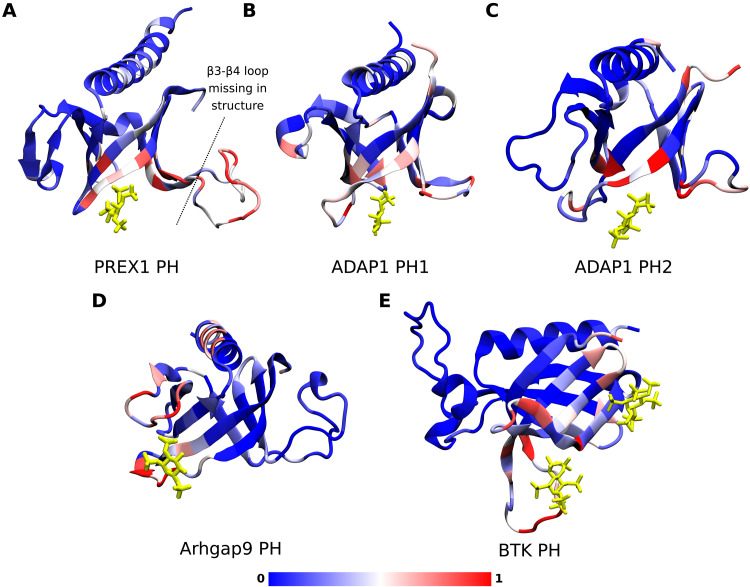
Comparison of simulated phosphoinositide interactions and crystallographic binding sites. Structures of the PH domains of (**A**) PREX1, (**B**) ADAP1 (PH1), (**C**) ADAP1 (PH2), (**D**) Arhgap9, and (**E**) BTK in which each residue is colored according to the normalized number of contacts observed between the protein and PIP_2_ and PIP_3_ headgroups during the final 200 ns of simulation, averaged over 20 replicates. Normalization was carried out by dividing the number of contacts at every residue by the maximum number of contacts that any residue in that PH domain made with PIP headgroups. The position of the bound PIP headgroup analog in the Protein Data Bank (PDB) file of each structure is also shown in yellow stick representation (PDB IDs: 5D3X, 3LJU, 2P0H, and 4Y94).

The capability of CG-MD simulations to identify crystallographic phosphoinositide binding sites on PH domains and other proteins has been previously demonstrated (*42*, *43*). In this section, we compare the results of our simulations for three PH domains with known crystallographic PIP binding sites that have not been simulated in previous studies—the two PH domains of ADAP1 and the PREX1 PH domain, which are all known to have canonical binding sites. In addition, we discuss an example of a PH domain with a noncanonical crystallographic inositol binding site (Arhgap9) and one with multiple crystallographic PIP binding sites (BTK). Analysis of the number of contacts that each residue made with PIP_2_ and PIP_3_ headgroups during the final 200 ns of simulation and comparison with the relevant crystal structures (Fig. 2) shows that our simulations correctly predicted the binding of a phosphoinositide headgroup in the binding site suggested by the crystal structures for these PH domains. We note that our contact analysis also identified additional interaction sites on the exterior of the β-barrel structure of these PH domains.

In the structure of the human PREX1 PH domain [Protein Data Bank (PDB) ID: 5D3X] in complex with inositol-(1,3,4,5)-tetrakis phosphate at the canonical site, examination of the side chains within 4 Å of the ligand shows that residues R289, R328, K368, K280, and Y300 are engaged in electrostatic and/or hydrogen-bonded interactions with the phosphates in the 3, 4, and 5 positions. S282 and Q287 also lie within 4 Å of the ligand and stabilize the interaction (*15*). During our simulations, we find that all these residues, except Q287, have contacts with PIP headgroups at a frequency of at least 70% of that of the residue with the most contacts. Furthermore, K280 and R289 are the residues in the canonical binding site that make the most contacts with PIP headgroups in the simulations, which is consistent with experimental findings using differential scanning fluorimetry that these are the residues that are most important for PIP_3_ binding to the canonical pocket (*15*). In addition to binding at the canonical site, our analysis for the PREX1 PH domain also reveals substantial interaction of phosphoinositide headgroups with the disordered β3-β4 loop, which is rich in basic residues and has been modeled in for the simulations as it is absent from the structure. Experimentally, it has been shown that mutations that abolish binding to the canonical site do not abolish membrane binding in the PREX1 PH domain but that membrane association is substantially reduced by deletion of β3-β4 loop residues 311 to 318 (*15*). This has led to a model of membrane interaction and activation in which nonspecific electrostatic interaction between anionic lipids and the β3-β4 loop drives membrane association and thus allows PI(3,4,5)P_3_ binding to the canonical site, which then allosterically activates PREX1 (*15*). Our contact analysis captures both of these key interaction sites.

Similarly, we observe regions of high PIP contacts defining the canonical binding pockets of both PH domains of ADAP1. In contrast, for the Arhgap9 PH domain, which lacks a canonical binding site, we do not observe these but instead we observe high numbers of contacts along outward facing residues of the β1 strand and β1-β2 loop, as well as the β5-β6 loop, defining the atypical binding pocket observed in the crystal structure. In addition, we see high numbers of contacts along the face of the C-terminal helix that has a cluster of basic residues aligned along the putative membrane-binding interface. These nonspecific interactions with anionic lipids potentially stabilize the membrane-bound orientation of the protein. Lastly, for the BTK PH domain, we observe high numbers of PIP contacts at both the canonical and atypical binding sites, which were observed in the BTK PH/PIP headgroup crystal structure. Additional interactions are along the β3-β4 loop, which may constitute a third interaction site. Overall, our contact analysis captures both interaction sites, again demonstrating the power of our simulation method to reproduce crystallographic binding sites while adding detail of key interactions that are absent from some structures. Beyond the examples described here, we searched the PDB for all structures of PH domains with crystallographic evidence of inositol phosphate binding sites and found 18 such cases—comparison between the simulations and crystallographic evidence is presented in fig. S4 for those 18 PH domains. Contact analysis for all 100 simulated PH domain structures is presented in fig. S5.

**Fig. 3.**
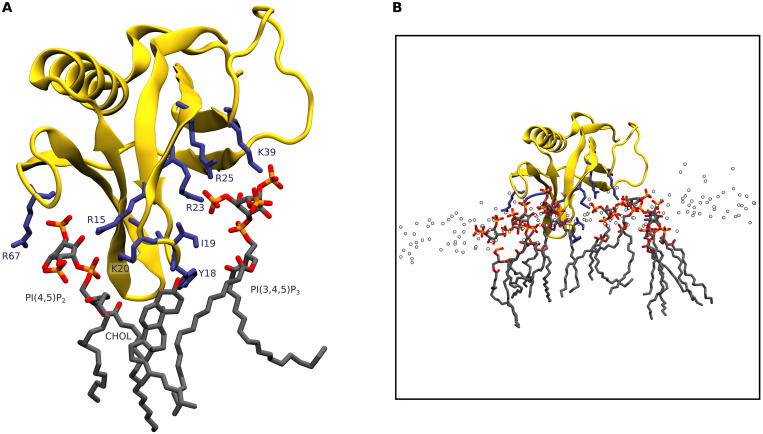
An atomistic model of membrane-bound Akt1 PH after backmapping and 200 ns of atomistic simulation. (**A**) Geometry of PI(3,4,5)P_3_ bound in the canonical site, with PI(4,5)P_2_ bound in a putative noncanonical site on the opposite face of the β1-β2 loop, meanwhile the hydrophobic tip of the loop inserts into the membrane and engages in hydrophobic interactions with cholesterol and lipid acyl tails. (**B**) The membrane-bound state involves association of multiple anionic lipids with the PH domain.

To examine these interactions in more detail for one PH domain, Akt1, the end point of one simulation, in which PIP_3_ was bound to the known canonical site, was backmapped to an atomistic representation and simulated for a further 200 ns using the CHARMM36 force field (*25*). The interactions within the binding pocket (Fig. 3) are similar to the crystal structure, with R23, R25, and K39 engaged in electrostatic and hydrogen-bonded interactions with the position 3 and position 4 phosphates. In addition, we find that the hydrophobic tip of the β1-β2 loop formed by Y18 and I19 inserts into the membrane and engages in hydrophobic interactions with an acyl tail of the bound PIP_3_ and a cholesterol molecule. The β2-β3 and β3-β4 loops face away from the membrane, as has previously been suggested (*25*). In addition to interactions with the canonically bound PIP_3_, the membrane-binding interface is lined with basic residues that facilitate association with eight additional PIP_2_, PIP_3_, and PS lipids in the simulation snapshot (Fig. 3). In particular, there is a PIP_2_ bound in the pocket formed between the β1-β2 and β5-β6 loops (similar to the noncanonical binding site seen in the Arhgap9 PH domain), which is stabilized by interaction with R15, K20, and R67. Previous work has shown that R15 and K20 are critical for binding of the Akt1 PH to PS containing liposomes, and we propose that the other basic residues lining the membrane-binding interface are likely to also contribute toward stabilization of the Akt1 PH domain on the membrane, involving multivalent interactions with anionic lipids (*35*).

### The β1-β2 region provides the primary site of PIP contacts in most PH domains

**Fig. 4.**
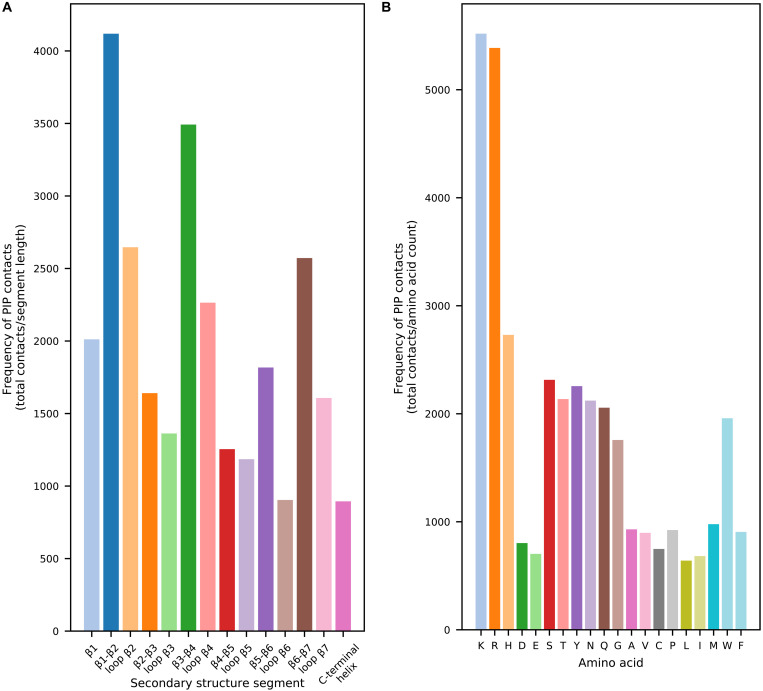
Secondary structure and amino acid contributions to phosphoinositide contacts. (**A**) Frequency with which each of the secondary segments of PH domains made contacts with phosphoinositide headgroups during all simulations. PH domain residues were assigned to 1 of 14 conserved secondary structure units, and contact frequency was calculated by summing contacts for each residue assigned to the secondary structure unit over all PH domains and simulation replicates and dividing by the total number of residues assigned to that secondary structure unit. (**B**) Frequency with which each amino acid type contacted PIP_2_ and PIP_3_ headgroups during all simulations. Frequency was calculated by summing contacts for the amino acid over all simulation replicates of all PH domains and dividing by the total number of occurrences of that amino acid in the simulated sequences.

To obtain a global view of PIP–PH domain interactions, we examined the contribution to phosphoinositide headgroup contacts during simulations from each of the conserved secondary structure segments found in PH domains, allowing us to establish patterns and exceptions in the phosphoinositide contact profile across the family. Assigning the residues of the simulated PH domains to 1 of 14 secondary structure segments (seven strands, six interstrand/loop regions, or the C-terminal helix), we totaled the PIP headgroup contacts of each segment during the final 200 ns of all simulations. To determine the frequency of contacts at each segment, we normalized to also take into consideration the sequence length of the segments. For the purpose of this analysis, the short, structured regions sometimes found between the classical PH domain strands have been assigned to the loop between the strands. This analysis (Fig. 4A) reveals that the β1-β2 loop is the segment most likely to interact with PIPs, followed by the β3-β4 loop, β2 strand, and β6-β7 loops. The importance of the β1-β2 loop has long been known, but our study also showed substantial interactions of the β3-β4 loop and β6-β7 loops. These loops form a triad at the base of the β-sheets in PH domains with canonical binding sites, but the very high number of contacts of β3-β4 loop and β6-β7 loops also suggests association of multiple PIP lipids with the PH domains during the simulations. A similar analysis can be applied at the amino acid level, highlighting the importance of cationic lysine, arginine, and histidine residues for stabilizing PIP headgroup interactions (Fig. 4B).

**Fig. 5.**
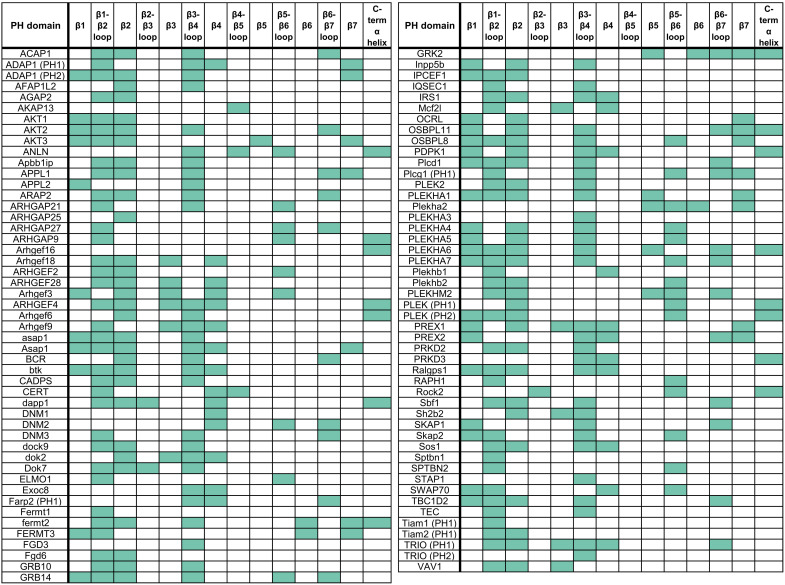
Identification of structural segments with substantial contributions toward PIP interactions for individual PH domains. Summary of simulated PH domain contacts with phosphoinositide headgroups, identifying whether each of the 14 conserved structural elements of the PH domain had a residue with normalized contacts above a threshold value of 0.8. Those with contacts in a segment above the threshold are colored green otherwise white. Some PH domains that could not be reasonably assigned to the classical PH domain secondary structure pattern were omitted for this analysis (see Materials and Methods).

We next examined the contact frequency of secondary structure segments for individual PH domains. Residue-level contacts with PIP_2_ and PIP_3_ headgroups were totaled during the final 200 ns of simulation for each PH domain and normalized by dividing the maximum number of contacts made by a single residue in that PH domain. To reduce the complexity of the analysis, we selected a normalized contact frequency of 0.8 as a threshold for a residue with substantial contributions toward PIP headgroup interactions in the PH domain, as this threshold captured the key residues for known crystal binding sites. Using this threshold, we determined whether each secondary structure segment contains a residue that contributes substantially to PIP interactions (Fig. 5).

Using this analysis, we found that 85% of the analyzed PH domains [including those lacking the canonical KX_n_(K/R)XR motif in this region] have substantial PIP interactions at a residue in β1, β2, or the connecting β1-β2 loop. Consistent with our global analysis, this indicates functional conservation of the β1-β2 region for PIP binding. Furthermore, among those PH domains with the primary contact site in the β1-β2 region, 89% of those have additional contacts of similar frequency at alternative sites such as β3-β4 and β6-β7, pointing to the supplementary role of these loops in stabilizing the primary PIP binding site and/or in interacting with additional PIPs.

**Fig. 6.**
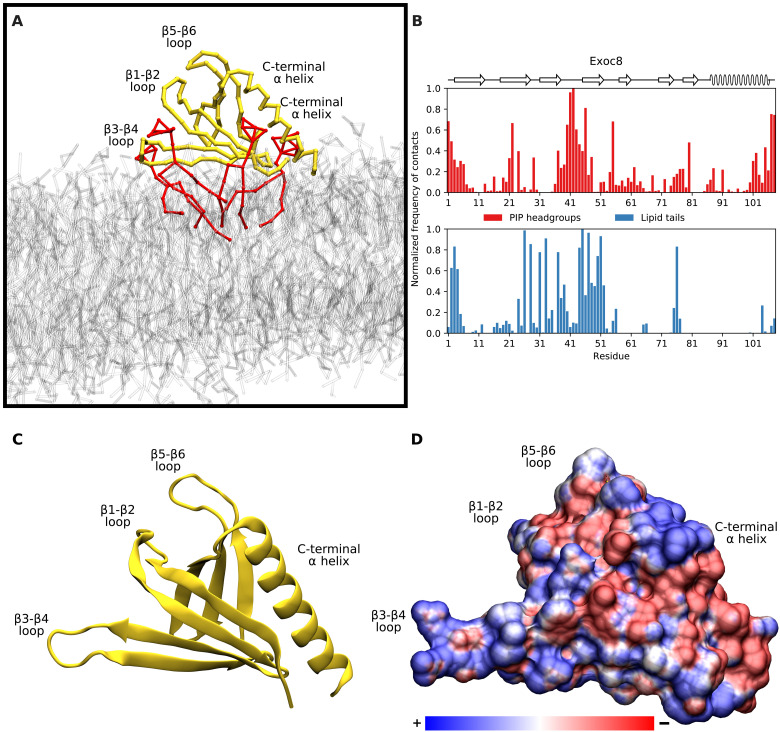
Interactions of the Exoc8 PH domain with the membrane. (**A**) Simulation snapshot showing the preferred membrane-bound orientation of Exoc8 PH. The lipid bilayer is shown in gray, with the PIP lipids that associated with the PH domain shown in red. (**B**) Normalized number of contacts between the Exoc8 PH domain and PIP headgroups (red) or lipid tails (blue) reveals a preference for phosphoinositide interaction with the β3-β4 loop and not the β1-β2 loop. (**C**) Atomistic ribbon structure of the Exoc8 PH domain (PDB ID: 1ZC3), in the same orientation as in (A). (**D**) Electrostatic potential map of Exoc8 PH in the same orientation as (A) and (C), demonstrating the electropositive β3-β4 region.

This systematic analysis of the location of PIP contact sites also reveals interesting exceptional cases that do not conform to the interaction patterns discussed above. Below, we discuss in more detail the results for the Exoc8 PH domain that is one such example, in which the β3-β4 loop is the primary contact site observed for interactions with phosphoinositide headgroups (Fig. 6). This PH domain lacks basic residues in the β1-β2 loop, which are key for electrostatic interaction with anionic PIP headgroups. Instead, the primary site for PIP interaction is formed by a pair of arginines at the tip of the β3-β4 loop. These electrostatic interactions are supplemented by insertion of hydrophobic residues spread along the membrane-interacting interface from β2 to β4. Examination of the electrostatic profile of the Exoc8 PH domain reveals a long electropositive ridge on one side of the β-barrel, arising from β3, β4, and their connecting loop. This electropositive ridge is not mirrored on the opposite β1-β2 side of the barrel, and we find that the Exoc8 PH domain stably adopts a “side-on” membrane-bound orientation, maximizing the contact between the positive ridge and the negative membrane surface. Exoc8 has recently been found to bind specifically to PI(4,5)P_2_, although there is currently no experimental insight into its mechanism of membrane association (*11*).

### Association of multiple PIPs with PH domains

**Fig. 7.**
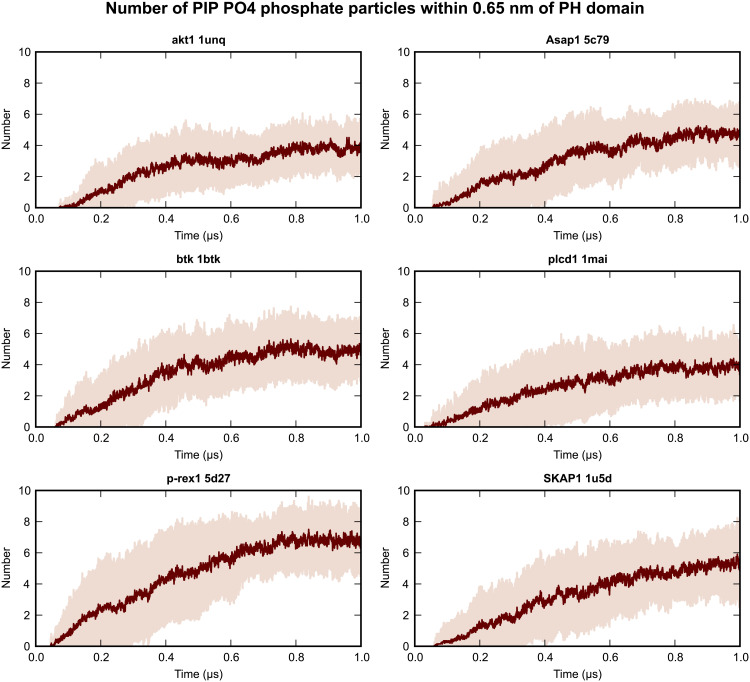
Multiple phosphoinositide molecules associate with PH domains during simulation. Plots of the number of PO4 (CG representation of position 1 phosphate) particles of PIP_2_ and PIP_3_ lipids within a 0.65-nm cutoff distance of the PH domain during simulations. The mean of 20 simulations for the AKT1, Asap1, btk, plcd1, p-rex1, and SKAP1 PH domains is plotted in dark red, with pale red shading representing the interval ±1 SD of the mean. The data for all simulated PH domains are shown in fig. S6.

**Fig. 8.**
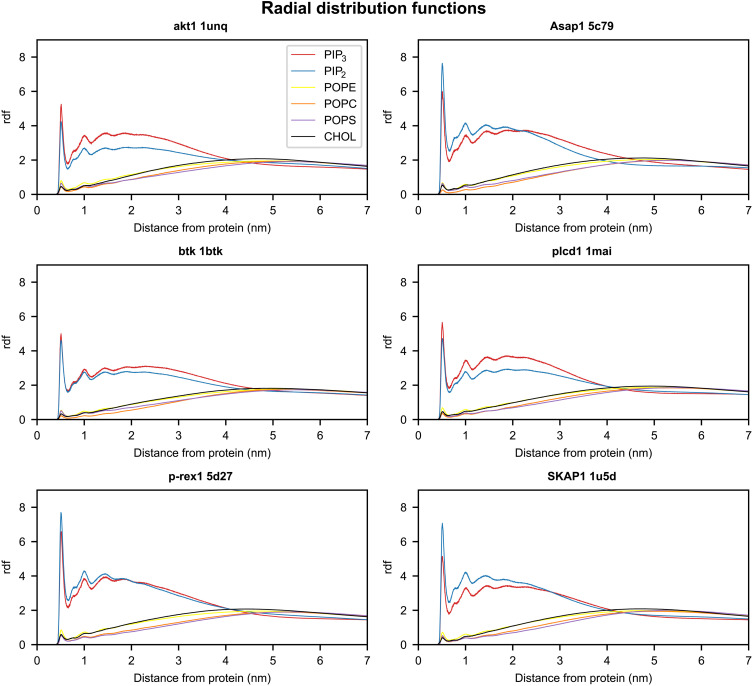
Lipid radial distribution functions demonstrate clustering of PIP lipids around PH domains. Radial distribution functions for all lipid species (PIP_2_, red; PIP_3_, blue; cholesterol, black; POPS, purple; POPE, yellow; POPC, orange) during the final 200 ns of simulation of all replicates for the AKT1, Asap1, btk, plcd1, p-rex1, and SKAP1 PH domains. The data for all simulated PH domains are shown in fig. S8.

For all simulated PH domains, we observed that after initial binding to the bilayer, multiple PIPs are recruited and closely associate with the PH domain (Fig. 7). In the final 200 ns of simulation (using 0.65-nm cutoff distance to the PO4 phosphate particle), most PH domains have at least four PIPs within the cutoff distance (fig. S6). For Arhgef18, DNM2, and SKAP1, we also extended the simulations to 2 μs to examine whether the clustering will be retained (fig. S7). Our results suggest that the average number of PIPs associated with these PH domains at 2 μs is very similar to the number at 1 μs. Furthermore, clustering of PIP lipids in the vicinity of PH domains induces modifications to the local lipid environment, as seen through analysis of the lipid radial distribution function during the final 200 ns of simulation (Fig. 8 and fig. S8). Other recent computational studies that have examined multiple phosphoinositide binding to PMPs have used total phosphoinositide compositions ranging from 5 to 10% (*31*, *32*, *34*, *43*–*45*). Our model membrane has concentrations of PIP_2_ (7%) and PIP_3_ (3%) that are at the upper end of this range. This PIP-rich model may bias the simulation toward multiple PIP binding. To test this possibility, we repeated the simulations at lower PIP concentrations (3% PIP_2_ and 1% PIP_3_) for the Plcδ1 PH domain. Similar multiple PIP association with the PH domain was observed at this lower PIP concentration after 2 μs of simulation (fig. S9). This shows that while clustering may take longer with a different composition, the phosphoinositide concentration is not biasing our observation of phosphoinositide clustering.

### PH domains adopt diverse membrane orientations

**Fig. 9.**
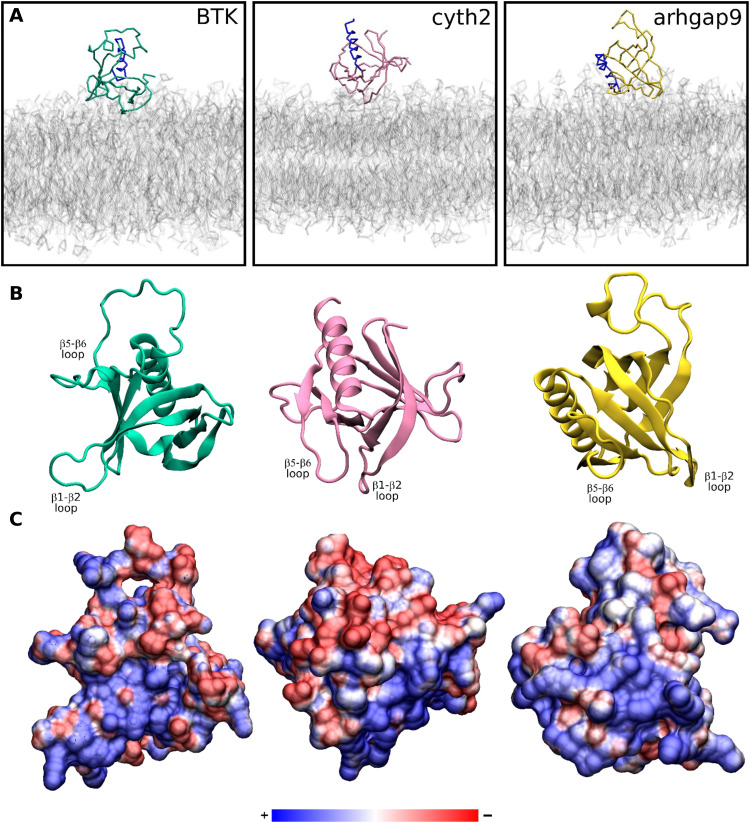
Diversity of preferred membrane-bound orientations of PH domains. (**A**) Simulation snapshots of the preferred membrane-bound orientations of the BTK (cyan), cyth2 (pink), and arhgap9 (yellow) PH domains. The C-terminal α helices are shown in blue to highlight the orientational differences. These orientations correspond to the densest state of the distance–rotation density matrices shown in fig. S10. (**B**) Atomistic ribbon structures of the three PH domains, shown in the same orientation as in (A). (**C**) Electrostatic potential surfaces of the three PH domains, shown in the same orientation of as in (A) and (B).

Lastly, we examined the orientations of PH domains on membranes. Distance–rotation density matrices (fig. S11), which are two-dimensional (2D) histograms of protein-membrane distance versus protein orientation observed during the simulations, demonstrate that most PH domains adopt a preferred orientation on the membrane. Some PH domains, such as the dynamin family members (DNM1, DNM2, and DNM3), can adopt multiple distinct orientations on the membrane. Furthermore, the orientational preferences can differ between PH domains, depending on how the electrostatic profile and anionic lipid binding sites are distributed about the β-barrel. To illustrate this, in this section, we discuss three example PH domains that adopted quite different preferred orientations on the membrane. The PH domains of BTK (PDB: 1btk) and Cyth2 (PDB: 1u29) have crystal structures with inositol phosphate binding at the canonical site, whereas binding to the atypical binding site between the β1-β2 and β5-β6 loops is seen in the crystal structure of arhgap9 (PDB: 2p0h). BTK preferentially associated in an orientation in which all loops except β5-β6 are in contact with the membrane, and the canonical PIP binding site is occupied. The β5-β6 loop in this PH domain is rich in glutamic acid residues and points away from the membrane surface due to electrostatic repulsion (Fig. 9). In contrast to BTK, the PH domain of arhgap9 adopts a side-on orientation, with the positively charged face of the barrel containing the β1-β2, β4-β5, and β5-β6 loops in contact with the membrane. The β2-β3 and β3-β4 loops point away from the membrane. This orientation of the arhgap9 PH domain enables phosphoinositide headgroup binding to the atypical site formed between the β1-β2 and β5-β6 loops. The PH domain of Cyth2 adopts an orientation that is intermediate between that of BTK and arhgap9. It does not have the negatively charged β5-β6 loop that maintains the BTK PH domain in an “upright” orientation. Its electrostatic potential map is asymmetric around the barrel, leading to a side-on orientation, but it is not quite as asymmetric as arhgap9. We observe phosphoinositide contacts with the β5-β6 loop in Cyth2 (fig. S10), suggesting a noncanonical PIP binding site that is not observed in the crystal structure. However, the normalized frequency of contacts in this region is less than in arhgap9. This comparison suggests that electrostatics are important in determining the interaction with anionic lipids and the orientation of the PH domain on the membrane.

## DISCUSSION

Using high-throughput CG-MD simulations, we have systematically compared the membrane interactions of 100 mammalian PH domains. We have observed that the β1-β2 region in PH domains is the primary site that makes contacts with PIPs in most of the family members but, in most cases, is supplemented by interactions at other sites, particularly the β3-β4 and β5-β6 loops. The significance of the β1-β2 loop for phosphoinositide binding has been suggested previously for a number of PH domains in both canonical and atypical binding modes, and we show that while this is the primary site on average, it is typically supplemented by adjacent loops—such as the β3-β4 loop in the canonical binding mode and the β5-β6 loop in the arhgap9-like atypical binding site (*12*, *15*, *46*, *47*). Other regions have also been shown to play a role in phosphoinositide atypical binding in some PH domains, such as the β3 and β4 strands in the BTK PH domain and the β6-β7 loop in the CERK PH domain (*33*, *34*, *48*). Furthermore, we show that the Exoc8 PH domain is an exceptional case, which binds primarily through its β3-β4 loop.

Following initial membrane binding, additional PIPs are recruited, eventually saturating between four and six PIPs closely associating (<0.65 nm) with the typical PH domain. This multiple PIP association occurs even when using membrane compositions with lower (4%) total phosphoinositide concentrations. There is a growing body of evidence that membrane association by individual PH domains involves interaction with multiple PIPs, background anionic lipids such as POPS, or other lipid types such as sphingolipids (*10*, *15*, *30*–*32*, *37*, *49*, *50*). Multiple binding sites for PIPs or soluble phosphoinositides have been identified for the ASAP1, BTK, PLEKHA7, dynamin, and acap1 PH domains (*30*, *32*–*34*, *50*, *51*). Enhancement of membrane binding by the presence of background anionic lipids such as POPS has been observed for the AKT1 and ASAP1 PH domains (*31*, *35*). These multiple interactions have been proposed to influence the orientation, localization, affinity, and diffusivity of membrane-associating proteins (*31*, *52*–*54*). Our work systematically ties the findings from these individual studies together and shows that the capacity for interaction with multiple PIPs and PH domain–induced lipid clustering are general properties of membrane-associating mammalian PH domains, consistent with large-scale studies of cooperative lipid binding in yeast PH domains (*10*).

The relative strength and specificity of the multiple interaction sites remain to be determined. Local lipid modulation has been previously observed in simulations of integral membrane proteins, for which the altered local lipid environment provides a unique fingerprint (*55*). Our simulations suggest a similar behavior for PH domains. The PH domains may create a unique fingerprint in the membrane enriched with anionic lipids, which may regulate their interactions with partner proteins. Alternatively, recognition of already existing PIP clusters in the membrane could localize them to particular membrane regions or to the vicinity of an integral membrane protein interaction target.

It is important to consider some limitations of our methodology. We have used CG-MD simulations that use some approximations in modeling of both the protein and the lipids. Despite these approximations, the consistency of our findings with much of the recent literature on individual PH domains suggests that these simulations have the capacity to identify rather accurately the regions of important lipid interactions, including those that have not been captured in structural studies; although recent experiments are revealing some of these additional interactions, they remain difficult to identify at the molecular level systematically (*31*, *32*). In addition, our simulation method does not adequately sample membrane-unbinding events that would enable us to determine the thermodynamics and relative affinities of PH domain binding to our model membrane system. The preferred binding orientations in our simulations can, however, provide a starting point for the use of biased simulation methods, such as the generation of a potential of mean force using umbrella sampling, that would allow calculation of the strength of the PH domain–membrane association (*56*). Furthermore, although the CG model captures the preference that PH domains have for interaction with PIPs over other lipid types, the resolution of the model is not sufficient to accurately capture the experimental specificity that some PH domains display for PIP phosphorylation levels or positional isomers; hence, our analysis focuses on PIP_2_/PIP_3_ interaction in general. However, PH domain specificities and binding affinities have been extensively studied, and experimental techniques enable these to be characterized systematically (*11*, *57*).

Understanding the protein-lipid interactome is crucial to improve our understanding of membrane protein structure, function, and pharmacology, but it remains largely uncharacterized due to the diversity and complexity of membrane lipids and limitations in experimental techniques (*41*, *58*). The increasing realism and throughput of CG-MD simulations are poised to allow systematic characterization at the molecular level. There are a few examples in which the lipid interactions of multiple proteins in complex bilayers have been systematically simulated in a single study (*43*, *59*–*61*). Furthermore, the MemProtMD database contains simulations of over 5000 integral membrane structures in a single-component 1,2-dipalmitoyl-sn-glycero-3-phosphocholine (DPPC) lipid bilayer (*62*). The present work, covering 100 proteins and 2 ms of aggregate simulation time, demonstrates the growing power of high-throughput simulations to systematically study protein-lipid interactions and to investigate the patterns and differences in lipid binding within families. Our study demonstrates that it is feasible to systematically and realistically study protein-lipid interactions in complex bilayers for 100 proteins or more and identify lipid interaction sites consistent with experimental evidence. Constructing such large simulation datasets is an exciting approach, which, assisted by developments in machine learning, could facilitate a rapid expansion in our knowledge of the protein-lipid interactome at the molecular level.

While there is some work on individual domains, it is often challenging to experimentally study the molecular and mechanistic details of binding of these domains to membranes due to the dynamic nature of these events and the presence of the membrane. This study provides both a global picture of the interaction of PH domains with the membrane and details of the interactions of 100 individual domains with lipids. Our results show that the β1-β2 region in PH domains is the primary site that makes contacts with PIPs in most of the family members. However, in most cases, it is supplemented by additional interactions, particularly at the β3-β4 and β5-β6 loops. Furthermore, we show that there are some exceptions, e.g., the Exoc8 PH domain that binds primarily through its β3-β4 loop. Our results also demonstrate that PH domains can adopt different membrane-bound orientations and importantly induce clustering of anionic lipids. This clustering changes their local lipid environment which may be important for their function, as this may regulate their interactions with partner proteins. Finally, this study is a demonstration of how MD simulations can be used in high-throughput fashion to produce large datasets for protein-lipid interactions. Such data can be used to extract a global picture of the interactions of a family of proteins with lipids.

## MATERIALS AND METHODS

### Discovery, selection, and processing of PH domain structures for simulation

UniProt advanced websearches were conducted and cross-referenced with the PDB to find all reviewed PH domain containing sequences, with available PDB structures for human, rat, or mouse. This produced a list of 115 PH domains. The unusual “split” and “BEACH-type” PH domains of, for example, NBEA, Plcg1, and Snta1 were subsequently excluded as unsuitable for simulation and comparison with other PH domains. PH domains were chosen for simulation ad hoc. Where a protein contained two distinct PH domains, each PH domain was simulated independently. Structures were selected on the basis of resolution, low number of missing residues, and the absence of mutations. Selected PDBs were downloaded and processed for simulation. Processing involved extracting the PH domain from the rest of the structure and truncating two to four residues before the first PH domain β strand and two to four residues after the C-terminal α-helix to have a consistent structure for all the PH domains simulated. MODELLER was used to restore unresolved atoms or residues to the PH domain structure and to mutate any residues deviant from the wild-type UniProt sequence (*63*). Typically, only a few missing residues needed to be remodeled in the unstructured loop regions. Electrostatic potential maps of PH domain structures were generated using the PDB2PQR and APBS tools, at pH 7 with the CHARMM force field (*64*).

### CG-MD simulations

Simulations were performed using GROMACS 5.0.7 and the Martini 2.1 force field (*65*, *66*). An automated script was developed and used to quickly and consistently build and equilibrate the simulation system and generate run files for production simulations. Processed PH domain PDB structures were converted to a CG representation using the martinize tool provided by the Martini developers and placed in a 16.5 nm by 16.5 nm by 20.5 nm simulation pbc box (*66*). The insane tool was used to add ions (0.1 M Na^+^ and Cl^−^) and solvent water and construct a symmetric membrane bilayer (both leaflets composed of 10% POPC, 40% POPE, 15% POPS, 7% PIP_2_, 3% PIP_3_, and 25% cholesterol) at a *z* distance of 7 to 8 nm from the protein (*67*). An elastic network model with a 0.7-nm cutoff distance was applied to protein backbone particles to constrain secondary and tertiary structure (*68*). All systems were energy-minimized and then equilibrated in the NPT ensemble for 2 ns with protein backbone particles restrained. For each system, 20 production simulations were run for 1 μs, with each repeat simulation initialized with random velocities according to a Maxwell-Boltzmann distribution. The LINCS algorithm was used to constrain bonds to equilibrium length (*69*). The velocity rescaling method was used to maintain a temperature of 323 K, with a 1-ps coupling time. Semi-isotropic Parrinello-Rahman coupling was used to maintain a pressure of 1 bar using a 12-ps coupling time (*70*, *71*).

### Atomistic MD simulations

The final frame of the 19th replicate simulation of the Akt1 PH domain was selected for backmapping to an all-atom representation due to the presence of PIP_3_ in the known canonical binding pocket. Backmapping of the system from Martini to the CHARMM36 force field was achieved using the backward method and the initram.sh script provided by the Martini developers (*72*). To correct for any structural changes within the protein during the CG simulation and backmapping, the backmapped protein coordinates were replaced with those from the original crystal structure (PDB ID: 1unq) after superimposition of the 1unq structure upon the backmapped structure using the confrms GROMACS command. CHARMMGUI provides parameters for several different PIP_2_ and PIP_3_ isomers—here, we used POPI25 and POPI35, which have a proton on the position 5 phosphate, on the basis of ab initio calculations, indicating that this is the most stable protonation state of PI(4,5)P_2_ and the evidence that Akt1 does not form strong interactions with the position 5 phosphate of inositol tetraphosphate (*25*, *73*). To ensure the correct headgroup stereochemistry of the PIP_3_ bound at the canonical site after backmapping, the headgroup coordinates of this POPI35 were replaced (after superimposition by rmsconf) by those of a reference POPI35 obtained from a pure POPI35 membrane constructed using the CHARMM-GUI membrane builder (*74*). The backmapped system was subsequently energy-minimized and subjected to 1 ns of equilibration in the NPT ensemble with the protein backbone restrained. An unrestrained production simulation was run for 200 ns, with a 2-fs time step, a temperature of 323 K, and a semi-isotropic Parrinello-Rahman pressure coupling at 1 bar.

### CG-MD analysis

The protein was first centered in the trajectory using gmx trjconv to prevent artifacts in analysis arising due to periodic boundary conditions. Root mean square deviation (gmx rms) and root mean square fluctuation (gmx rmsd) of protein backbone particles were calculated for each trajectory relative to its first frame. *Z*-axis distance between protein and membrane centers was calculated using the gmx dist command. Analysis of protein orientation was achieved by finding the rotation matrix (gmx rotmat) describing the transformation between a reference orientation and the orientation at each frame. The reference orientation was arbitrarily selected as the orientation in the end frame of the first simulation in the set of replicates. A Python script was developed for generating orientation density plots. These were constructed by taking the z-distance and the *R*_zz_ component of the rotmat data, correcting the rotmat to account for the membrane symmetry (*R*_zz_ value at a frame is multiplied by −1 if the z-distance at that frame is negative), and then generating and plotting a 2D histogram in matplotlib.

Contacts between all residues and lipids were calculated for the following lipid groups: POPC headgroup, POPE headgroup, POPS headgroup, POP2 headgroup, POP3 headgroup, all CHOL particles, POP2 and POP3 headgroups combined, and all lipid tails. gmx mindist was used to calculate whether molecule-possessing particles belonging to the given lipid group were within a 5.5 Å cutoff distance of any protein particles belonging to each residue at each simulation frame during the final 200 ns. One contact was counted at the residue for every frame in which a lipid particle in the group was within the cutoff distance. For each PH domain and each lipid group, contact counts at each residue were totaled across the 20 repeat simulations and then normalized by dividing the total contacts at each residue by the total contacts made by the residue with the highest number of contacts with that lipid group. This gives the normalized number of contacts, in which the residue with the most contacts has the value of 1, and all other residues have the contacts normalized relative to this residue. Convergence analysis was carried out as above, using differently sized samples of simulation replicates.

To calculate the number of PIP lipids closely associated with the PH domain during the time course of the simulation, gmx mindist was used to calculate the number of PO4 particles (the connecting phosphate between the tail and headgroup) belonging to either POP2 or POP3 lipids within a 0.65 nm distance of any protein particles. The mean and SD were calculated over 20 simulation replicates for each PH domain. A larger cutoff distance was used here than for the contact analysis due to the extra distance between the headgroup phosphates and the connecting PO4 phosphate. Radial distribution functions were similarly calculated for each lipid species using gmx rdf using the final 200 ns of simulation from all 20 replicates.

### Family-wide comparison of contacts by amino acid or secondary structure

For the family-wide analysis of amino acid contacts with PIP headgroups shown in Fig. 4, we grouped all residues by their amino acid type and totaled the contact counts (for the POP2 and POP3 headgroup lipid group) across all PH domains and simulation replicates. Normalization was conducted by dividing the total contacts for each amino acid type by the number of incidences of that amino acid in all the simulated PH domain sequences. The secondary structure analysis was plotted similarly but with the contacts instead grouped according to the conserved PH domain structural segments. An in-house Python script was developed for this grouping, which uses the STRIDE secondary structure assignment program to assign PH domain residues to one of 14 secondary structure segments given their structure (*75*). Normalization of the contacts was conducted by dividing the total contacts of each segment by the number of residues assigned to that segment over all the PH domains.

The 14 secondary structure segments were as follows: β1 strand, β1-β2 loop, β2 strand, β2-β3 loop, β3 strand, β3-β4 loop, β4 strand, β4-β5 loop, β5 strand, β5-β6 loop, β6 strand, β6-β7 loop, β7 strand, and the C-terminal α-helix. Unstructured N-terminal residues before the first strand were assigned to β1 strand, and unstructured C-terminal residues were assigned to the C-terminal α-helix. Several PH domains have short, structured regions inserted between the conserved structural segments, which complicate this analysis; to handle these cases, we assigned the additional short helices or strands to the nearest loop region where reasonable through manual correction of the STRIDE file. Similarly, because of inaccuracies with STRIDE or structure resolution, there are gaps in the strands of some PH domains, and these were also corrected by this analysis where reasonable by manually assigning the appropriate residues to the correct strand in the STRIDE file. The PH domains for which the STRIDE file was manually edited for this analysis were as follows: akap13 (6bca), anln (2y7b), ARHGAP27 (3 pp2), arhgap9 (2p0h), Arhgef18 (6bcb), ARHGEF2_5efx, Arhgef3 (2z0q), Arhgef6 (1v61), Arhgef9 (2dfk), DNM3 (5a3f), FERMT3 (2ys3), inpp5b (2kig), PLEK PH1 (1pls), p-rex1 (5d27), PREX2 (6bnm), and sptbn1 (1btn). Three PH domains with additional structured regions that did not reasonably fit in with this analysis were excluded from this part of the analysis; these PH domains were as follows: ARHGEF1 (3odo), cyth2 (1u29), and cyth3 (1u29).

### Contacts threshold table

The residues of each PH domain (except ARHGEF, cyth2, and cyth3) were assigned to one of the 14 conserved structural segments as described above. For each segment in the PH domain, we used a Python script to determine (TRUE/FALSE) whether that segment contained a residue with normalized frequency of contacts with POP2 + POP3 headgroups above 0.8 (in other words, a residue with total contacts equal to at least 80% of the residue that had the most contacts in that PH domain). This gives a family-wide overview of which segments of each PH domain provide the key contribution to PIP interaction.

## Notes

View/request a protocol for this paper from *Bio-protocol*.
