# Supplementary material for: Systematic simulation of the interactions of pleckstrin homology domains with membranes
Source: Sci Adv. 2022 Jul 6;8(27):eabn6992. doi: 10.1126/sciadv.abn6992 (PMC9258823; doi:10.1126/sciadv.abn6992)
Supplement: Supplementary file 1 — Figs. S1 to S12 Table S1 [file sciadv.abn6992_sm.pdf]

Science Advances
AAAS

Supplementary Materials for

Systematic simulation of the interactions of pleckstrin homology domains with membranes

Kyle I. P. Le Huray *et al.*

Corresponding author: Antreas C. Kalli, a.kalli@leeds.ac.uk

Sci. Adv. **8**, eabn6992 (2022)
DOI: 10.1126/sciadv.abn6992

This PDF file includes:

Figs. S1 to S12
Table S1

Fig. S1.

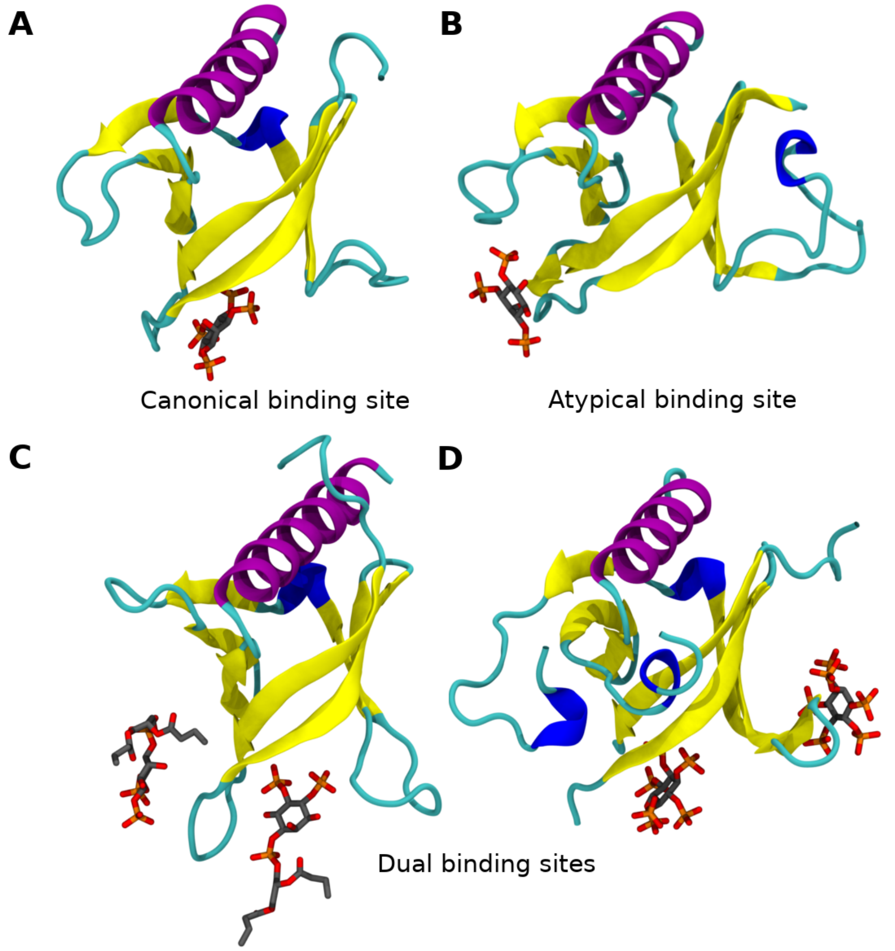

Fig. S1. Example canonical and atypical phosphoinositide binding sites observed in PH domain crystal structures. (A) Structure of the DAPP1 PH domain (PDB: 1fao), demonstrating inositol tetraphosphate bound at the canonical site. **(B)** Structure of the ArhGAP9 PH domain (PDB: 2p0h), with inositol trisphosphate bound at an atypical site on the outside of the barrel. **(C)** Structure of the Asap1 PH domain (PDB: 5c79), exhibiting dual binding of 04:0 PI(4,5)P_2 at both the canonical and an atypical site simultaneously. **(D)** Structure of the PH domain of BTK (PDB: 4y94), exhibiting dual binding of inositol hexakisphosphate to both the canonical site and an atypical site distinct from that of Asap1 or ArhGAP9.

Fig. S2.

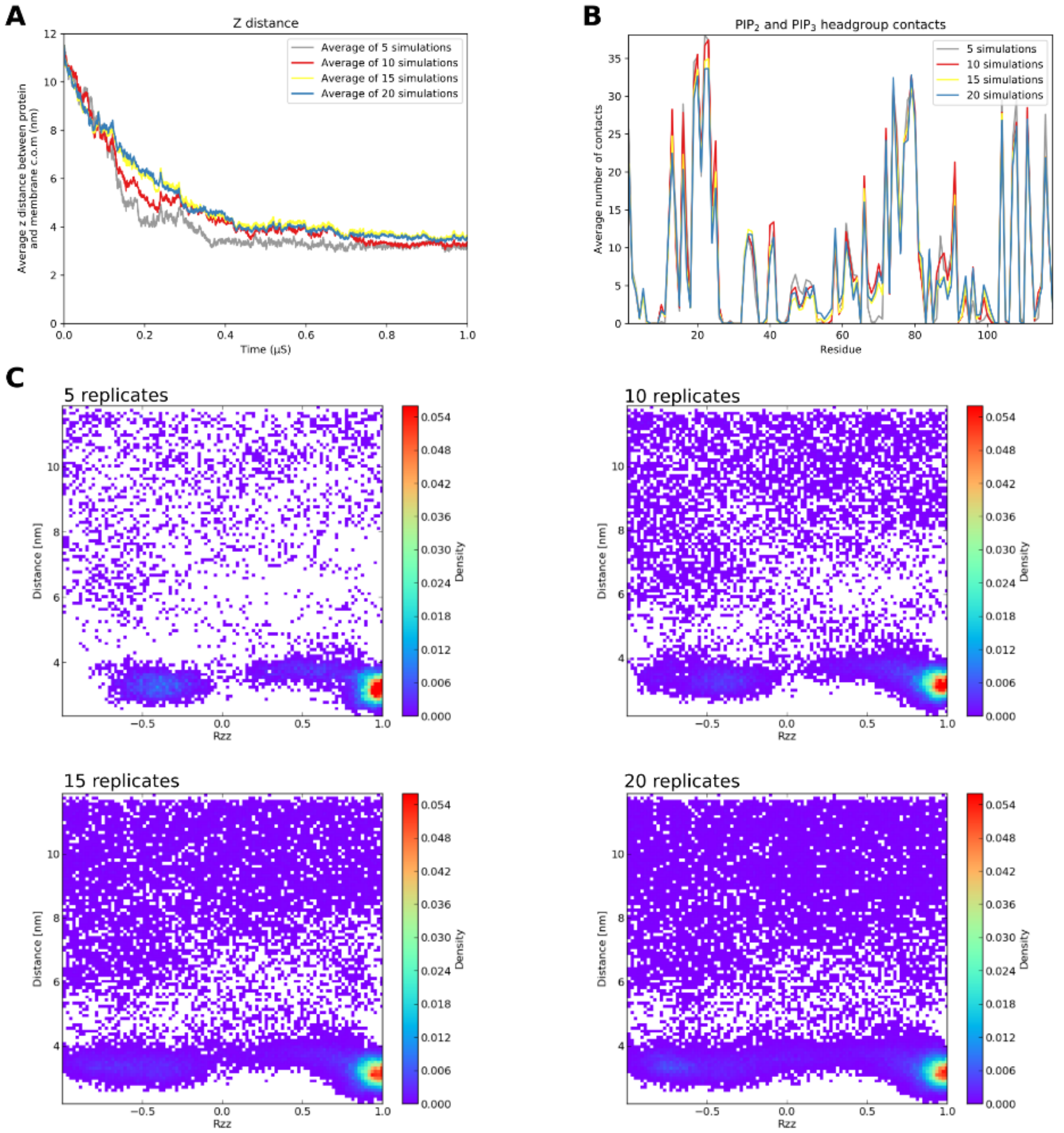

Fig. S2. Convergence of simulation data. Analysis of simulations for the BTK PH domain, using data from 5, 10, 15 and 20 replicates. 20 simulation replicates achieves convergence in: **(A)** protein-membrane z-axis distance over time, **(B)** contacts with PIP_2 and PIP_3 headgroups and **(C)** distance-rotation density analysis.

Fig. S3.

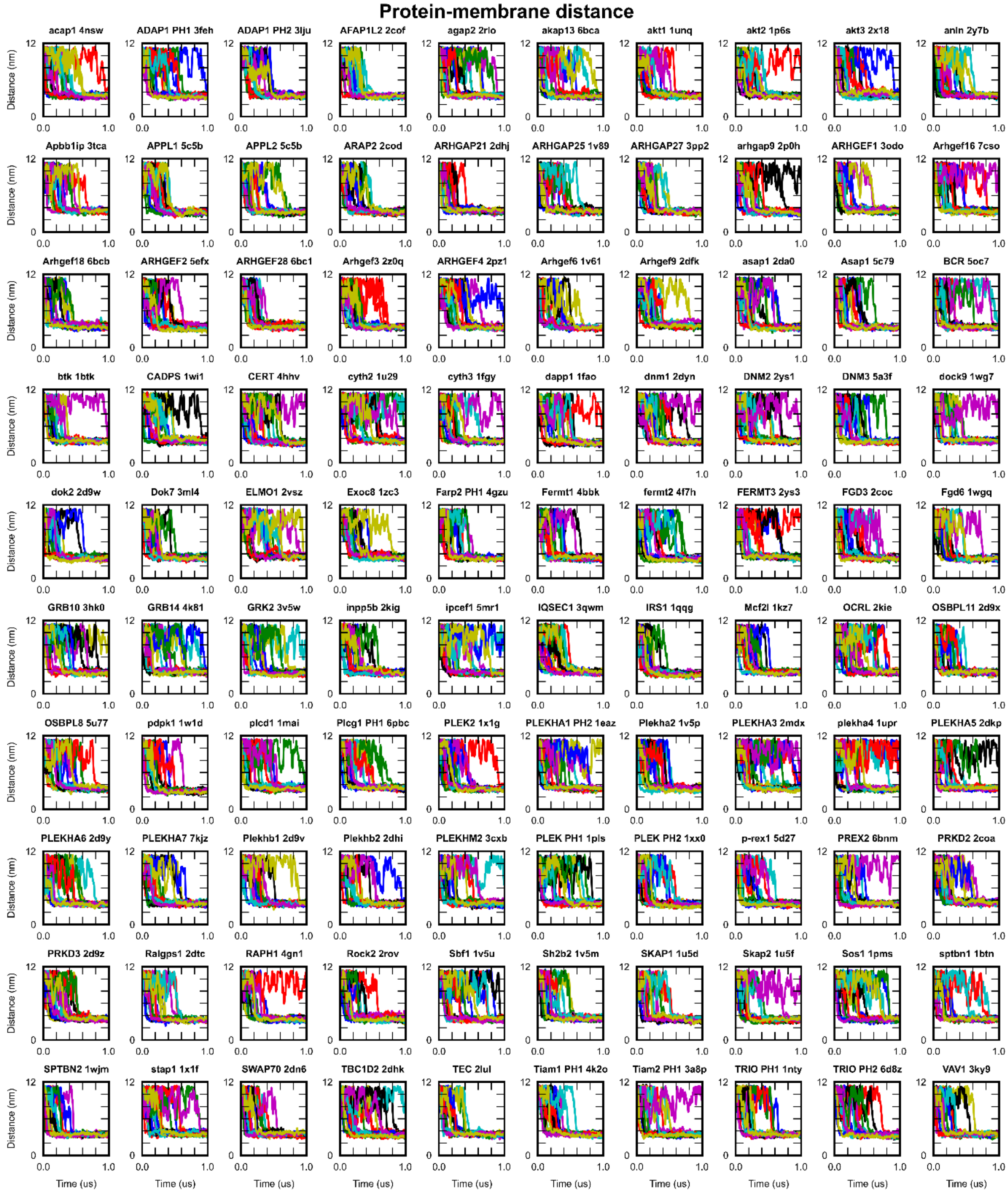

Fig. S3. Z-axis distance between protein and membrane centres of mass during the simulation time course. Data shown for all PH domain simulations, where each color represents the trajectory for an independent replicate. Membrane binding is observed at a distance of approximately 4 nm. Distances have been corrected to account for periodic boundary conditions.

Fig. S4.

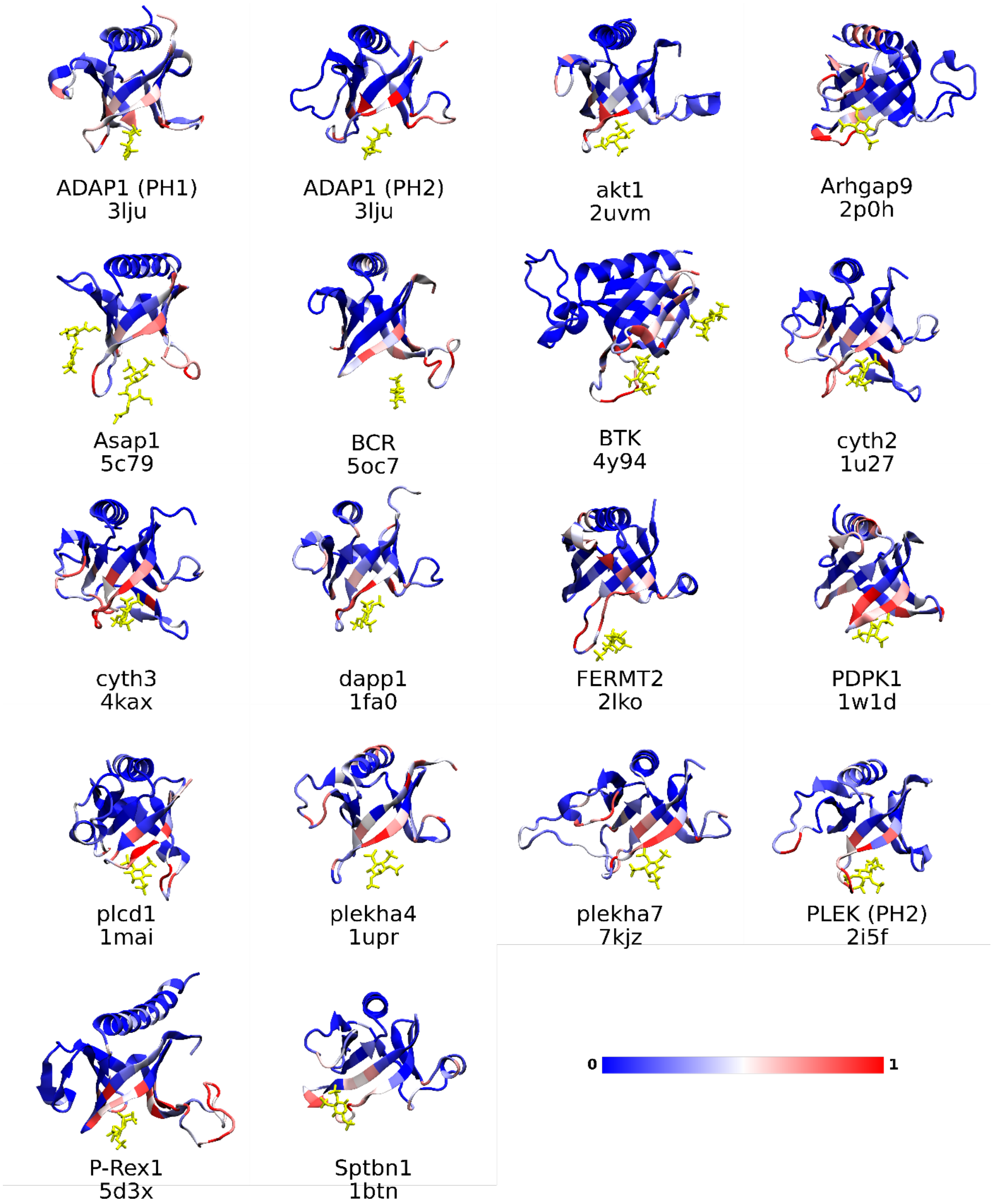

Fig. S4. Comparison of simulated phosphoinositide interactions and crystallographic binding sites for 18 PH domains with crystallographic inositol binding sites. Structures of 18 PH domains in complex with inositols (or analogues), in which each residue is colored according to the normalized number of contacts observed between the protein and PIP_2 and PIP_3 headgroups during the final 200 ns of simulation, averaged over 20 replicates. Normalization was carried out by dividing the number of contacts at every residue by the maximum number of contacts that any residue in that PH domain made with PIP headgroups. The position of the bound PIP headgroup

analogue in the PDB file of each structure is also shown in yellow stick representation. PDB IDs are shown below the protein name.

Fig. S5.

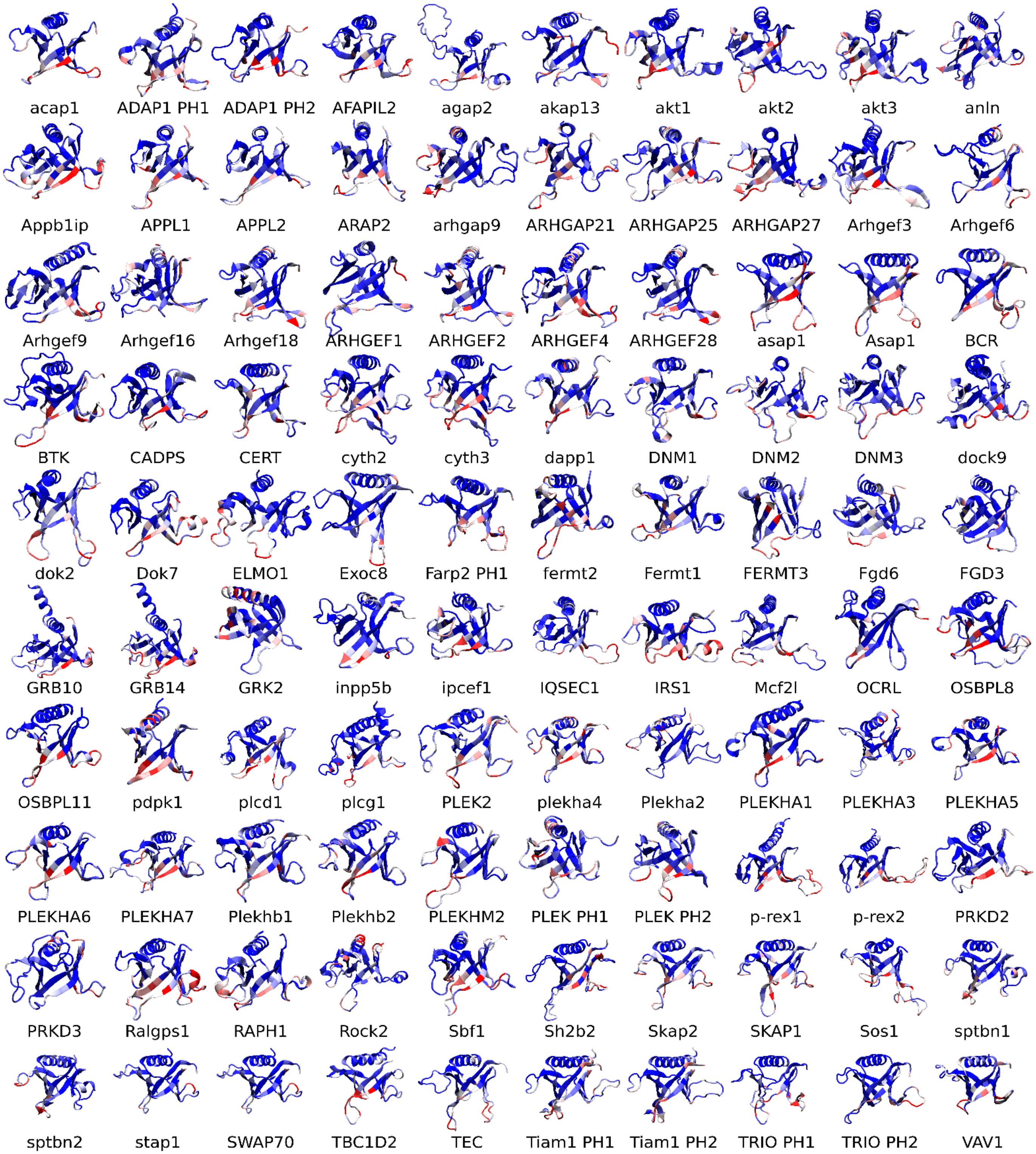

Fig. S5. Structural comparison of PIP headgroup interaction hotspots on all simulated PH domains. Structures of the 100 simulated PH domains where each residue is colored according to the normalized number of contacts observed between the protein and PIP_2 and PIP_3 headgroups during the final 200 ns of simulation, averaged over 20 replicates. Normalization was carried out by dividing the number of contacts at every residue by the maximum number of contacts that any residue in that PH domain made with PIP headgroups. The color scale is the same as in Fig. 2 and Fig. S4.

Fig. S6.

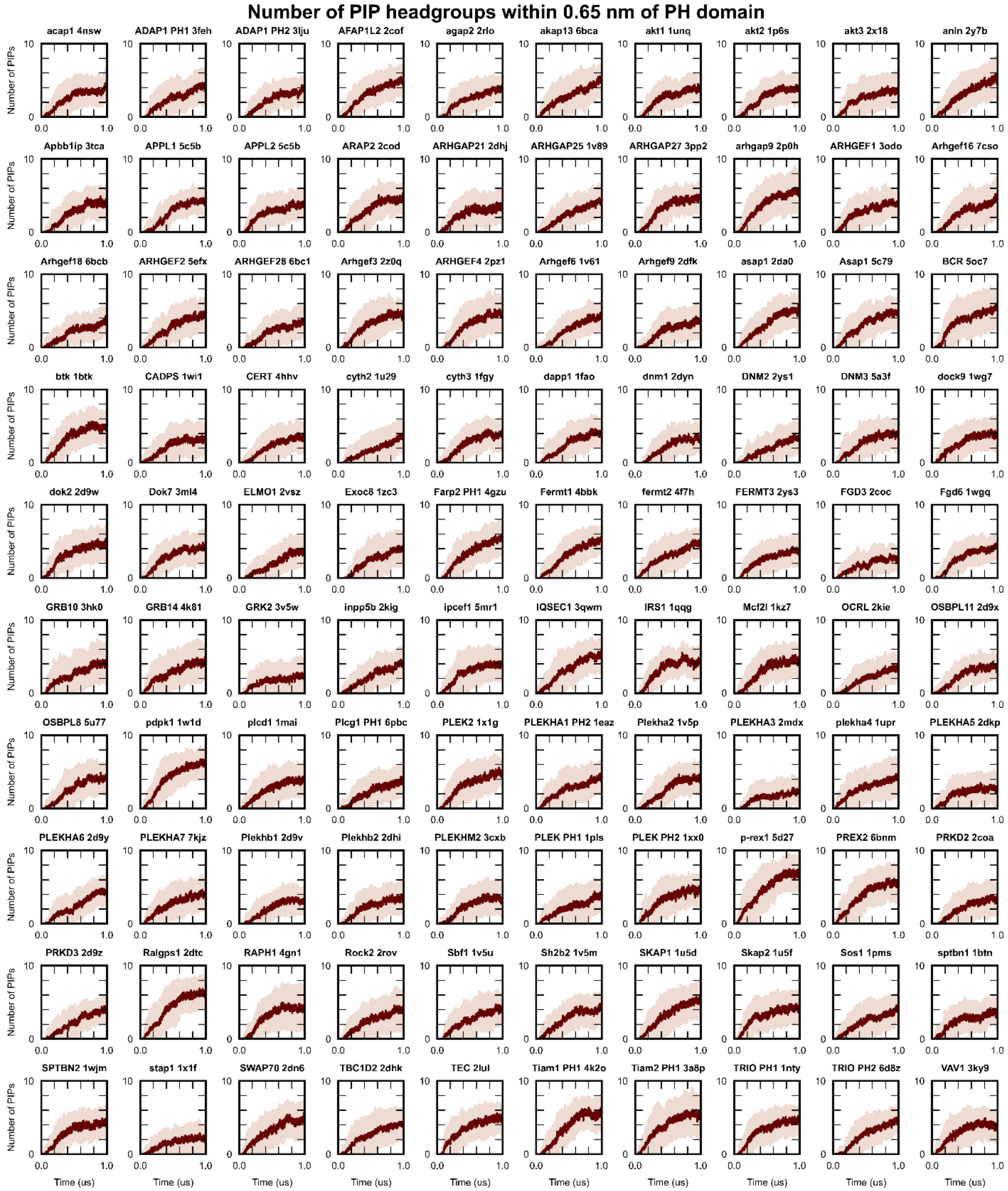

Fig. S6. Association of multiple PIP lipids with PH domains following membrane binding. The number of PO4 (CG representation of position 1 phosphate) particles of PIP_2 and PIP_3 lipids within a 0.65 nm cutoff distance of each PH domain during the course of simulations. The mean of 20 simulations is plotted in dark red, with pale red shading representing the interval +/- 1 standard deviation of the mean for every PH domain.

Fig. S7.

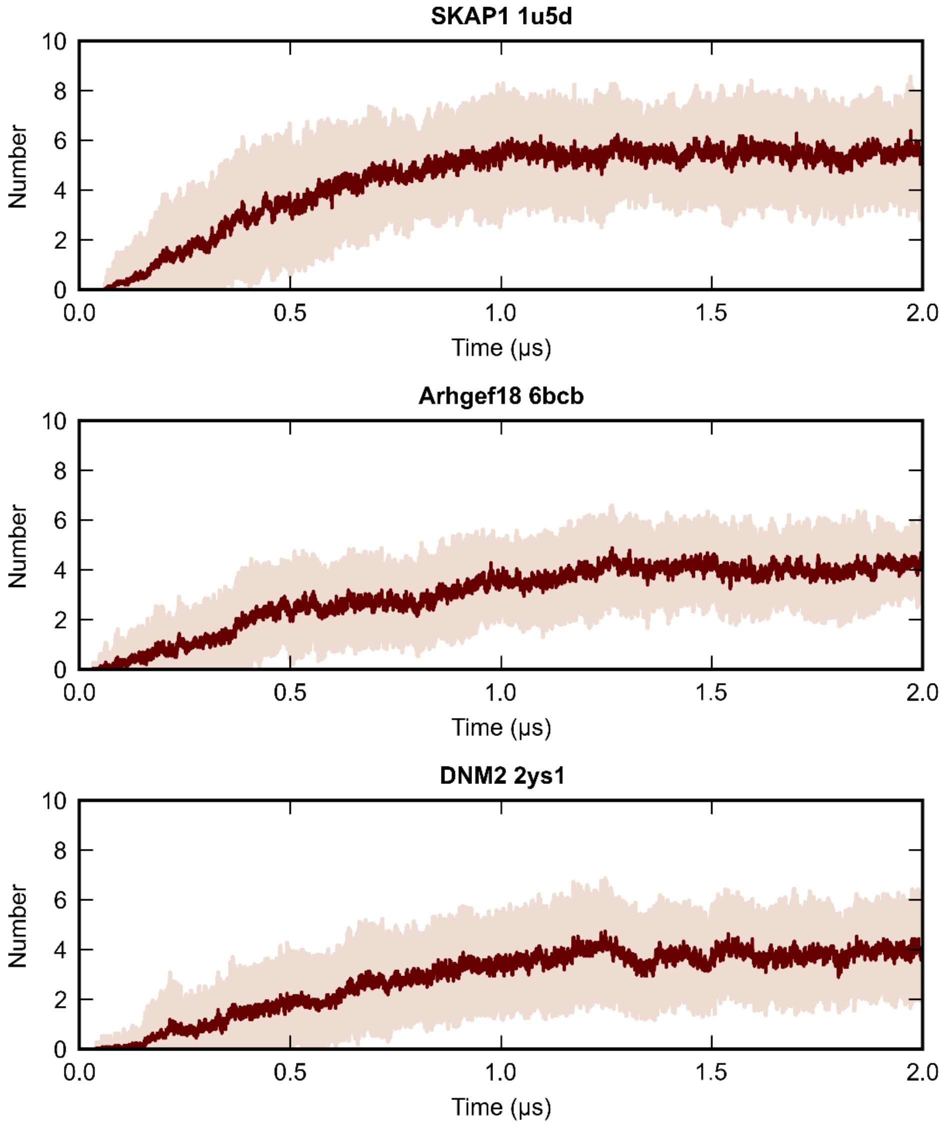

Fig. S7. Number of PIP lipids associate with SKAP1, Arhgef18 and DNM2 PH domains. The number of PO4 (CG representation of position 1 phosphate) particles of PIP_2 and PIP_3 lipids within a 0.65 nm cutoff distance of each PH domain during the course of simulations. The mean of 20 simulations is plotted in dark red, with pale red shading representing the interval +/- 1 standard deviation of the mean for every PH domain. The number of PIPs associated after 2 μs is similar to the number at 1 μs.

Fig. S8.

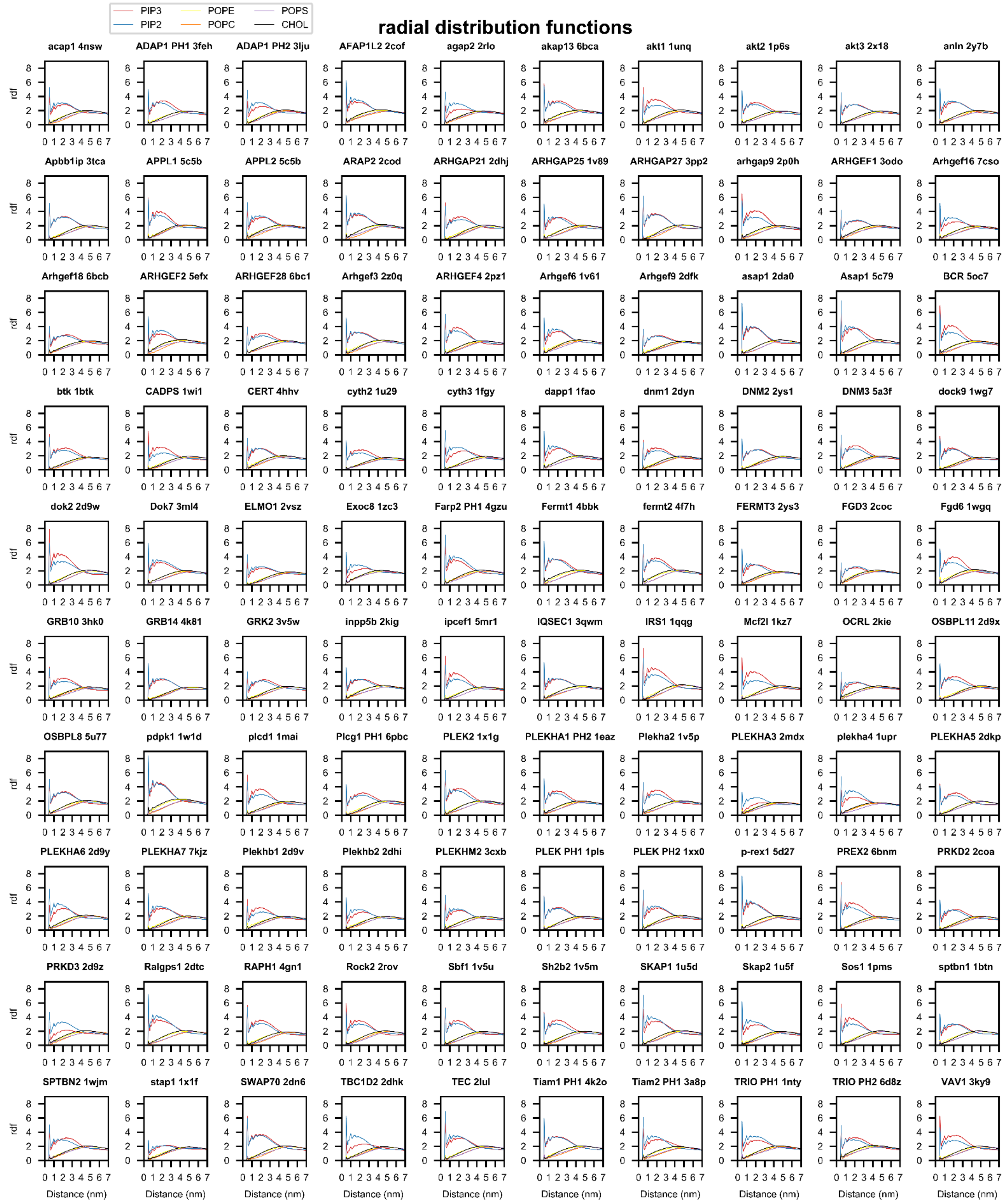

Fig. S8. Lipid radial distribution functions around each PH domain demonstrate clustering of PIP lipids. Radial distribution functions for the PO4 particles of each lipid species (PIP_2: red, PIP_3: blue, cholesterol: black, POPS: purple, POPE: yellow, POPC: orange) during the final 200 ns of simulation of all replicates for each PH domain for all simulated PH domains.

Fig. S9.

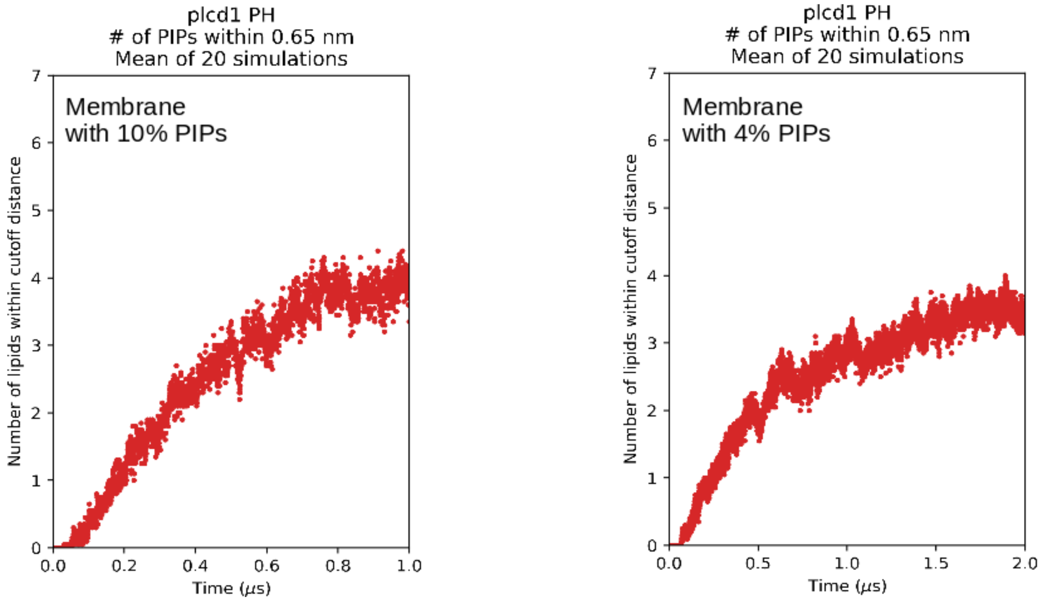

Fig. S9. Association of multiple PIPs with the plcd1 PH domain in membranes with 10% and 4% PIP composition. The number of PO4 (CG representation of position 1 phosphate) particles of PIP_2 and PIP_3 lipids within a 0.65 nm cutoff distance of the PH domain during simulations. The mean of 20 simulations is plotted in red.

Fig. S10.

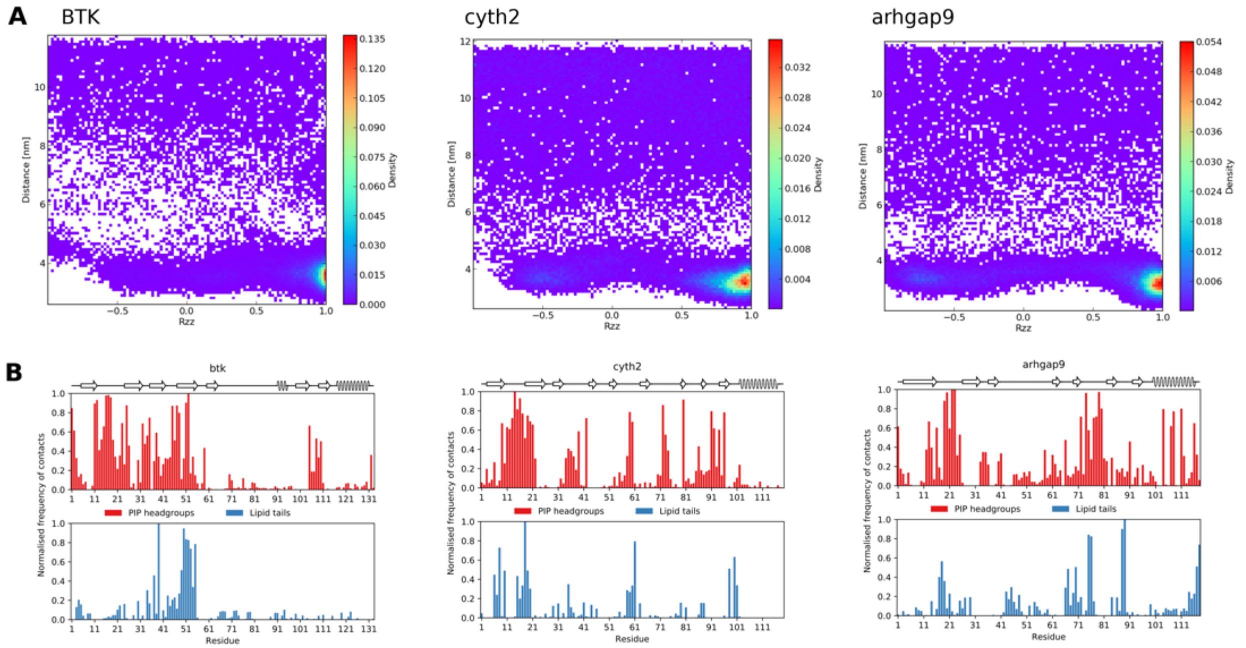

Fig. S10. (A) rotation-distance density matrices, and **(B)** normalized contacts between the protein and PIP_2 and PIP_3 headgroups (red), or phospholipid tails (blue) for the BTK, cyth2, arhgap9 PH domains.

Fig. S11.

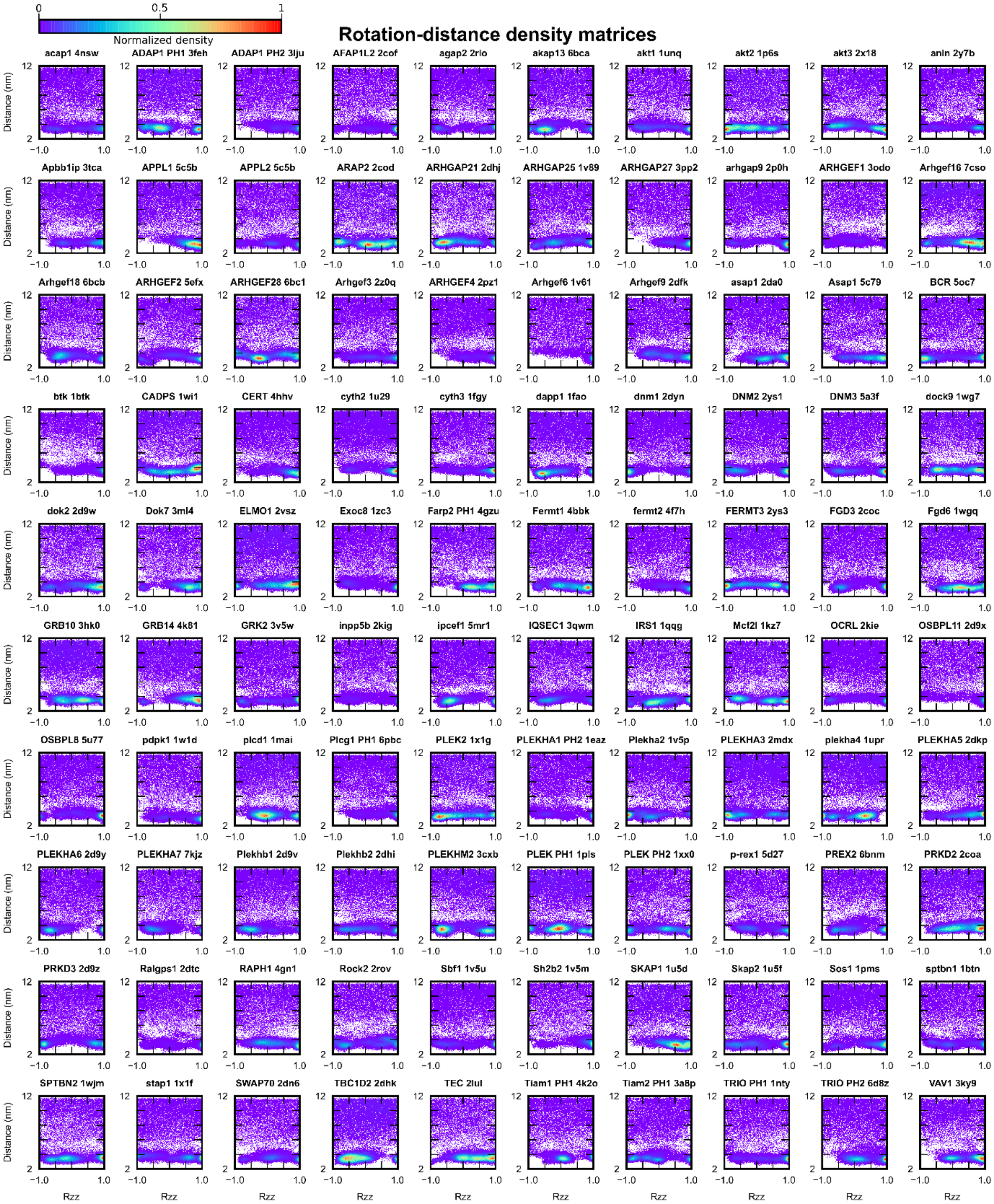

Fig. S11. PH domains adopt preferred orientations on the membrane, as demonstrated by rotation-distance density matrices. Each 2D histogram show the density of protein-membrane z-distance and orientation states observed during simulation for each PH domain. The orientation was characterized by the Rzz component of the rotation matrix describing the transformation between an arbitrary reference orientation and the PH domain at each frame, corrected for periodic boundary conditions. Reference orientations differ for each PH domain.

Fig. S12.

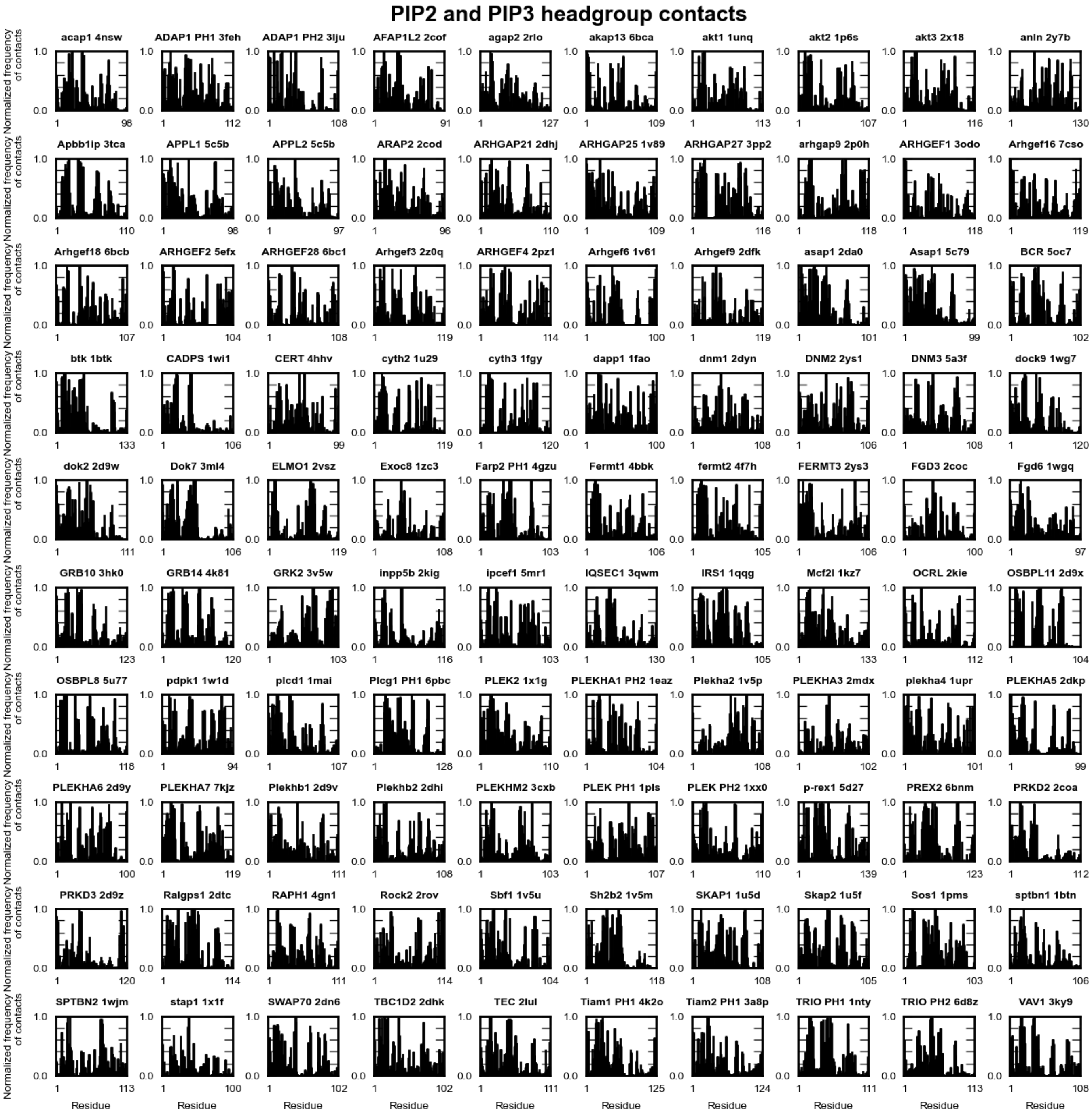

Fig. S12. Normalized contacts between PIP_2/PIP_3 headgroups and all residues of all PH domains. One contact was counted at a residue for every frame in which a PIP_2 or PIP_3 headgroup particle was within a 5.5 Å cut-off distance of any particles of the residue. Contacts were summed over 20 repeat simulations, and then normalized for each PH by dividing the total contacts at each residue by the total contacts made by the residue with the highest number of contacts in that PH domain.

Table S1.

PH domain	PDB ID of structure coordinates used for simulation
acap1	4nsw
ADAP1 (PH1)	3feh
ADAP1 (PH2)	3lju
AFAP1L2	2cof
agap2	2rlo
akap13	6bca
akt1	1unq
akt2	1p6s
akt3	2x18
anln	2y7b
Apbb1ip	3tca
APPL1	5c5b
APPL2	5c5b
ARAP2	2cod
ARHGAP21	2dhj
ARHGAP25	1v89
ARHGAP27	3pp2
arhgap9	2p0h
ARHGEF1	3odo
Arhgef16	7cso
Arhgef18	6bcb
ARHGEF2	5efx
ARHGEF28	6bc1
Arhgef3	2z0q
ARHGEF4	2pz1
Arhgef6	1v61
Arhgef9	2dfk
asap1	2da0
Asap1	5c79
BCR	5oc7
btk	1btk
CADPS	1wi1
CERT	4hhv
cyth2	1u29
cyth3	1fgy
dapp1	1fao
dnm1	2dyn
DNM2	2ys1
DNM3	5a3f
dock9	1wg7
dok2	2d9w
Dok7	3ml4
ELMO1	2vsz

Exoc8	1zc3
Farp2 (PH1)	4gzu
Fermt1	4bbk
fermt2	4f7h
FERMT3	2ys3
FGD3	2coc
Fgd6	1wgq
GRB10	3hk0
GRB14	4k81
GRK2	3v5w
inpp5b	2kig
ipcef1	5mr1
IQSEC1	3qwm
IRS1	1qqg
Mcf2l	1kz7
OCRL	2kie
OSBPL11	2d9x
OSBPL8	5u77
pdpk1	1w1d
plcd1	1mai
Plcg1 (PH1)	6pbc
PLEK2	1x1g
PLEKHA1 (PH2)	1eaz
Plekha2	1v5p
PLEKHA3	2mdx
plekha4	1upr
PLEKHA5	2dkp
PLEKHA6	2d9y
PLEKHA7	7kjz
Plekhb1	2d9v
Plekhb2	2dhi
PLEKHM2	3cxb
PLEK (PH1)	1pls
PLEK (PH2)	1xx0
p-rex1	5d27
PREX2	6bnm
PRKD2	2coa
PRKD3	2d9z
Ralgps1	2dtc
RAPH1	4gn1
Rock2	2rov
Sbf1	1v5u
Sh2b2	1v5m
SKAP1	1u5d
Skap2	1u5f

Sos1	1pms
sptbn1	1btn
SPTBN2	1wjm
stap1	1x1f
SWAP70	2dn6
TBC1D2	2dhk
TEC	2lul
Tiam1 (PH1)	4k2o
Tiam2 (PH1)	3a8p
TRIO (PH1)	1nty
TRIO (PH2)	6d8z
VAV1	3ky9

Table S1 – List of all simulated PH domains and the coordinate files used for simulation
